# Difficult Labour: A Guide to Its Management for Students and Practitioners

**Published:** 1894-09

**Authors:** 


*Difficult Labour: A Guide to its Management for Students and Practitioners.* By G. ERNEST HERMAN, M.B. Pp. xvi., 443. London: CASSELL & COMPANY, LIMITED. 1894.

This work is a sequel, or rather a supplement, to a handy and useful little book which was brought out about three years ago by Dr. Ernest Herman, the Senior Obstetric Physician to the London Hospital. That book was then intended to be a guide to medical students and also to midwives beginning obstetric practice. It gave them plain and practical directions how to manage ordinary cases of labour, and also clearly indicated the danger-signals which give warning of difficulties and complications, in which it will be their duty to call in the aid of a well qualified practitioner.

Though by no means a large book, the present work is a veritable *multum in parvo*, for it contains in a very moderate space a large amount of clear and precise information as to how these difficulties should be recognised and treated in the unavoidable absence perhaps of some well-skilled and experienced obstetrician. In such respects this very portable work is likely to be of value to young practitioners who have just started in some remote country district, where it is almost impossible to obtain such consultations. It will be likewise a useful help in refreshing the memory of a student who is just going up for his examination in obstetric medicine; for the author, from his own experience as an examiner, is likely to know well the wants of young men when about to undergo this trying ordeal.

The first chapter of Dr. Herman's book is very short, for it occupies a little over a page in space. It begins with a good definition of natural labour, and then insists upon the importance of *early* diagnosis of the child's position, because "abnormal presentations can easily be set right at the proper time, but are very difficult of correction when that time has passed." To do this, however, when there is scarcely any dilatation of the os uteri would require, we should say, an accoucheur whose hands have been well practised in abdominal palpation. Chapters II. and III. are rather longer, but still very concise considering the amount of useful matter contained in them; they treat of untoward head presentations, such as difficult occipito-posterior and face and brow presentations, and the best modes of altering these to more favourable ones, provided such attempts are made before the head becomes too deeply engaged in the pelvic cavity. Chapter IV. contains an excellent series of illustrations of that wise provision of nature called "the moulding of the head," which ensues in the unfavourable head presentations just mentioned, and very concise explanations of the head's shape and position are given. Chapter V. is much longer, and gives a good account of various difficulties in pelvic presentations and the manipulations which may be required to correct these, in all of which the hands of a skilful accoucheur are the best instruments to be used. We quite agree with the author when he says, "Have nothing to do with forceps for the breech"; but he admits, however, its occasional use to extract the after-coming head. We have never met with a case that required it; but should there be one of that kind, the instrument must be used very promptly, if the child's life is to be saved by it. In the chapters that follow, Dr. Herman treats of transverse presentations, of compound presentations owing to prolapse of extremities or of the umbilical cord, and then describes twin labours, labours rendered difficult by double monstrosity of the child, or diseases such as hydrocephalus, or tumours which increase its size, &c. Many rare and difficult cases, such for instance as the interlocking of the two heads or other parts of twins, and the appropriate treatment required in each case, are clearly though briefly described. The longest, the most complete, and elaborate part of the book, occupying six chapters and rather more than a hundred pages, is devoted to a description of the worst form of obstructed labour, viz., that which arises from deformed pelvis; the diseases and accidents which in the first place cause these deformities; the best mode of measuring the pelvis (viz., by the hand of the accoucheur and not by instruments); the instrumental assistance that will be required, ranging from the forceps to hysterotomy, &c. All this part of the work treats of a great deal that is new and original, and is interspersed with numerous good engravings of rare and extraordinary kinds of deformity, such as we seldom see represented in the ordinary text-books of midwifery. This is, perhaps, the best and most complete part of the book. The remaining chapters, from XX. to XXIV. inclusive, treat of various dangerous complications of labour, such as ruptures of the uterus, injuries of the genital canal, hemorrhages—accidental, unavoidable, and *post-partum*,—inversion of the uterus, etc. These are followed by Chapter XXV., consisting of nearly thirty pages, on "The Forceps," the proper use of which is very well described. This is followed by a short chapter on "Turning," in which the bi-manual method is well shown, both by description and some good illustrations. After this comes a chapter on "Craniotomy," which would, we think, come more appropriately immediately *after* that on the forceps. It describes the various "operations for lessening the child's size," and gives useful engravings of different instruments required for that purpose. Then we come to the *dernier ressort* of Midwifery, the Cæsarean section, and a short but clear account of an old friend with a new face; viz., Symphysiotomy. The concluding chapter, No. XXX., gives a useful substitute by means of which we may prevent some of these formidable operations; for it treats of "the induction of premature labour, and the best mode of performing it."

There is one dangerous complication of labour which is not noticed, and that is Puerperal Eclampsia. Perhaps the author was a little too scrupulous, and thought he could not fairly include it in a book which professes to treat strictly of "difficult labour." We regret the omission, for it rather mars the symmetry and completeness of his work. In the most dangerous cases of eclampsia, viz., those which come on in pregnancy when there is no sign whatever of labour, instruments are sometimes required for the purpose of inducing it; in eclampsia coming on during labour the forceps is often used to hasten delivery; and we also occasionally have recourse to a little instrument which many young men of the present day scarcely know how to use, that is, the lancet. We hope, therefore, to see this omission supplied in the second edition of this most useful guide for "students and practitioners."

